# Supplementary material for: The genome sequence of the Brassy Long-horn, Nemophora metallica (Poda, 1761) (Lepidoptera: Adelidae)
Source: Wellcome Open Res. 2025 Aug 15;10:445. [Version 1] doi: 10.12688/wellcomeopenres.24685.1 (PMC12504937; doi:10.12688/wellcomeopenres.24685.1)
Supplement: Peer review history

# Reviewer report: Reviewer response for version 1

**DOI:** 10.21956/wellcomeopenres.27201.r132335

**Published:** 2025-10-07

**Authors:** Christopher B. Cunningham

**Affiliations:** 1 University of Georgia, Georgia, USA

The project continues to produce quality resources for the insect genomics community. The report on the Brassy Long-horn, *Nemophora m*etallica, is no different. The methods are sound and relatively well documented. The article needs has enough details to be reasonably reproducible, especially now with the inclusion of the pipeline documentation. The data supporting the publication are publicly available.

 Abstract. There is no justification within the abstract, just results. At least a sentence that this was part of a large survey of insects is needed. And one sentence for its possible value.

 Keywords. These are repeated form the title and abstract so add no value. Google indexes off these too so they should reflect some broader value.

 Background. Background. The stated motivation for this study in the introduction is fine – part of a very large survey. It is short and too the point, gives some possible uses, and is very appropriate to the article type.

 Results. Table 1 is not really needed. They are not details that the reader needs to under the work and they can all be found under the NCBI BioProject ID or are repeated from the methods paragraph.

 BUSCO completeness needs to be assessed with the updated OrthoDB version 12. Difference from 10 to 12 have made a big difference for several insects I work with and colleagues have reported the same. The paper should not be indexed until this is updated. The new version has been for 6 months.

 Figure 4. Fig 4 has very little information beyond what is found elsewhere in the manuscript and can easily be summarized as a sentence in the results.

 Figure 3 & 5. Suggest some assembly dead-ends. But overall it seems very reasonable.  

 No genome annotation limits the use of the resource, but is not strictly needed.

Are sufficient details of methods and materials provided to allow replication by others?

Yes

Is the rationale for creating the dataset(s) clearly described?

Yes

Are the datasets clearly presented in a useable and accessible format?

Yes

Are the protocols appropriate and is the work technically sound?

Yes

Reviewer Expertise:

Insects, genetics, genomics, epigenetics, behavior, reproduction.

I confirm that I have read this submission and believe that I have an appropriate level of expertise to confirm that it is of an acceptable scientific standard, however I have significant reservations, as outlined above.

# Reviewer report: Reviewer response for version 1

**DOI:** 10.21956/wellcomeopenres.27201.r131299

**Published:** 2025-09-30

**Authors:** Frederique Hilliou

**Affiliations:** 1 SPE, Institut National de Recherche pour l'Agriculture l'Alimentation et l'Environnement Provence-Alpes-Cote d'Azur Site de Sophia (Ringgold ID: 60158), Sophia Antipolis, Provence-Alpes-Côte d'Azur, France

The publication of the genome of *Nemophora metallica* presents the life cycle and the host plant of this Adelidae in Finland, a country where this species is classified as endangered. *N metallica* like other members of Adelidae family has developed larval case-building behaviour. A chromosome-level genome containing 2 haplotypes was obtained from a male adult DNA extraction. It is scaffolded into 31 chromosomes including the Z-sex chromosome with an estimated haploid size of 708.66Mb. The manuscript also provides Merian elements on each chromosome which could be useful for comparative genomic studies within Lepidoptera. The used of the 32 Merian-elements could be proposed as a standard procedure in lepidopteran genome chromosome numbering. For instance, two Merian elements (M17 and M20) were fused on molecule 3 (OZ180157.1) as found in all Ditrysian (Wright et al 2024). (Ref 1)

 Tools are being developed to use proteins/CDS/transcript/RNAseq from closed species to obtain genome annotation. Unfortunately, no annotation is yet provided with this genome of *Nemophora metallica*, which will limit its use by biologist.

 Thy is the reason I choose "Approved with Reservations "

Are sufficient details of methods and materials provided to allow replication by others?

Partly

Is the rationale for creating the dataset(s) clearly described?

Yes

Are the datasets clearly presented in a useable and accessible format?

Partly

Are the protocols appropriate and is the work technically sound?

Yes

Reviewer Expertise:

plant insect interactions; insect detoxification

I confirm that I have read this submission and believe that I have an appropriate level of expertise to confirm that it is of an acceptable scientific standard, however I have significant reservations, as outlined above.

## References

[1] : Comparative genomics reveals the dynamics of chromosome evolution in Lepidoptera. *Nature Ecology & Evolution* .2024;8(4) : 10.1038/s41559-024-02329-4 777-790 10.1038/s41559-024-02329-4 38383850 PMC11009112

# Reviewer report: Reviewer response for version 1

**DOI:** 10.21956/wellcomeopenres.27201.r130134

**Published:** 2025-09-24

**Authors:** Arjen Van 't Hof

**Affiliations:** 1 Biology Centre of the Czech Academy of Sciences, Budějovice, Czech Republic

The genome assembly Brassy Long-horn is of outstanding quality, which we have become accustomed to. It is a bit challenging to come up with original comments after having done several of these genome assembly reviews. The quality is always close to perfect, and the methods seem so streamlined by now that it is hard to find any issues to comment on. Last week there was a very interesting article about dancing spiders on the BBC website, which ended by disclosing the aim to decode 10,000 genomes next year and complete all 1.8 million living species in the next decade. This would mean that, in case every genome is assessed by 2 reviewers, the editor would need to send out at least 20,000 review requests next year, and 3.6 million in the next decade. It could be that finding reviewers will become more challenging than producing genome assemblies at some point.

 The chromosome numbers are assigned based on Merian elements representing 32 ancestral chromosomes. I have not seen this in previous Lepidoptera genome assembly notes, which was surprising. I hope that standardizing the chromosome numbers based on ancestry as is done here for the Brassy Long-horn will become the norm for future assemblies.

 One minor comment:

 I assume the Nanodrop spectrophotometer was not used to assess the concentration of the sheared and purified DNA, but instead to establish the purity of the samples based on the 260/280 and 260/230 ratios.

Are sufficient details of methods and materials provided to allow replication by others?

Yes

Is the rationale for creating the dataset(s) clearly described?

Yes

Are the datasets clearly presented in a useable and accessible format?

Yes

Are the protocols appropriate and is the work technically sound?

Yes

Reviewer Expertise:

Lepidoptera genomics

I confirm that I have read this submission and believe that I have an appropriate level of expertise to confirm that it is of an acceptable scientific standard.
